# Analyzing support of postnatal transition in term infants after c-section

**DOI:** 10.1186/1471-2393-14-225

**Published:** 2014-07-11

**Authors:** Dimitrios Konstantelos, Sascha Ifflaender, Jürgen Dinger, Wolfram Burkhardt, Mario Rüdiger

**Affiliations:** 1 Department of Neonatology and Pediatric Intensive Care, Medizinische Fakultät Carl Gustav Carus, TU Dresden, Fetscherstraße 74, Dresden 01307, Germany

**Keywords:** Delivery room, Video recording, Transition

## Abstract

**Background:**

Whereas good data are available on the resuscitation of infants, little is known regarding support of postnatal transition in low-risk term infants after c-section. The present study was performed to describe current delivery room (DR) management of term infants born by c-section in our institution by analyzing videos that were recorded within a quality assurance program.

**Methods:**

DR- management is routinely recorded within a quality assurance program. Cross-sectional study of videos of term infants born by c-section. Videos were analyzed with respect to time point, duration and number of all medical interventions. Study period was between January and December 2012.

**Results:**

186 videos were analyzed. The majority of infants (73%) were without support of postnatal transition. In infants with support of transition, majority of infants received respiratory support, starting in median after 3.4 minutes (range 0.4-14.2) and lasting for 8.8 (1.5-28.5) minutes. Only 33% of infants with support had to be admitted to the NICU, the remaining infants were returned to the mother after a median of 13.5 (8-42) minutes. A great inter- and intra-individual variation with respect to the sequence of interventions was found.

**Conclusions:**

The study provides data for an internal quality improvement program and supports the benefit of using routine video recording of DR-management. Furthermore, data can be used for benchmarking with current practice in other centers.

## Background

During recent years, adequate scientific data became available to improve resuscitation of high risk or preterm infants in the delivery room. Subsequent recommendations are summarized in current resuscitation guidelines [1]. Yet, only a limited number of infants have to be resuscitated. Whereas the majority of newborns do not need any interventions some infants will present with a disturbed postnatal adaptation, and thus might require some medical support [2].

Infants delivered by cesarean section are well known to have an increased risk of disturbed postnatal adaptation [3-5]. Despite of an increasing number of deliveries by c-section [6,7], only limited data are available regarding medical care in these infants. However, for subsequent evidence based guidelines regarding medical support of postnatal transition more research on that issue is required.

The present study describes current DR-management of term infants born by c-section in our institution by analyzing videos that were recorded within a quality assurance program.

## Methods

Video recording was performed in a level 3 perinatal center with more than 2000 births per year as part of a quality assurance program [8]. The analysis was performed within that program and was approved by the local ethic committee (Ethikkomission an der Technischen Universität Dresden). Videos are stored in a way that recordings cannot attribute to an individual patient. Thus, no demographical data of individual patients are available and no parental consent was required for the present analysis.

### DR-management

In our institution residents are required to have a NLS-training once a year. Furthermore, the resident receives an one month “bedside” training on delivery room management by an experienced neonatologist. Thereafter, a pediatric resident or neonatal fellow takes care of low-risk infants born by planned c-section. In case of an emergency, a neonatologist is always available in close distance to the delivery room.

The general policy in our institution is spinal anesthesia for all planned c-sections. After birth, midwife places the newborn under a radiant heater. Newborns are placed on the resuscitation table with an umbilical cord clamping scissor. This will be changed with an umbilical cord clamp. According to local guidelines, infants should be dried and oxygen saturation will be measured. If infant’s condition is evaluated as appropriate, it will stay until 10 minutes after birth (in order to complete Apgar score by a delivery-independent caregiver) and will be returned to the mother thereafter [9]. If infant is considered to require medical support, appropriate interventions are provided including sustained inflations, which receive depressed term newborns with a heart rate <80 bpm. According to our protocol duration for every sustained inflation should be 10 seconds with a maximum of 3 sustained inflations. Sustained inflations should be performed in the first minutes of resuscitation. If the newborn show no improvement then the resuscitator should continue the resuscitation process using PPV. The pressure used for a sustained inflation is 20 cm H_2_O and for PPV 6-8 cm H_2_0. If support leads to an adequate improvement, infants return to their mother. If infant requires prolonged interventions in the NICU, an IV-line is placed in the delivery room, blood is collected and a glucose infusion is started. Thereafter infants are transferred – after a short visit to the mother – to the NICU.

### Video recording

Video recording of DR-management is performed as previously described [8]. For the present study, all recordings of planned c-section of term infants born in 2012 were analyzed. Since it was our aim to analyze only support of postnatal transition, we excluded videos of infants that had to be intubated or suffered from congenital malformation requiring immediate intensive care.

Analysis started with the arrival of the newborn under the radiant heater (Timepoint 0), which is usually about 20 seconds after cord clamping. For the current analysis we distinguished between infants with or without support of postnatal transition. In infants without any medical support, analysis ended either at transfer to the mother or after 10 minutes (whichever came first). In infants with support, analysis was extended until transfer to the mother or to the NICU.

### Analysis of data

#### Routine care

For all interventions performed to assess infant’s condition (including: auscultation, new towel to prevent heat loss, placement of saturation sensor, shortening of the umbilical cord with a clamp, temperature measurement) time point of first occurrence and duration were analyzed.

In infants with support of transition the following medical interventions were analyzed with regard to starting time, total duration and frequency of occurrence.

#### Respiratory support

CPAP administration via face mask, sustained inflations, ventilation with consecutive inflations, suctioning, CPAP-administration via naso-pharyngeal tube (which are routinely used for transfer to the NICU).

#### IV-line placement

In infants receiving an IV-line, time point and duration (defined as time between skin disinfection and stabilization of the IV-line with a band) of successful and unsuccessful IV-line placement attempts were determined.

#### Others

Time was measured in which the infant was absent from radiant heater (to be shown to the parents, to be weighted etc.). Furthermore, total time of no manipulations was calculated, with manipulations defined as every of the above described interventions or any contact of either medical personnel or father with infant’s skin.

### Statistical analysis

Descriptive data are presented as median and range. Interventions differed with respect to time of first occurrence, duration and frequency. A box-plot graphic was used to display variance with respect to time point of first occurrence. In addition, for each intervention the total (in seconds) and relative duration (percentage of total analyzed time) was calculated and displayed as median and range in a table.

### STROBE statement

We confirm that our research has adhered to the STROBE guidelines.

## Results

186 videos of DR-management in term infants born by c-section were analyzed, equaling 25% of all c-sections and 37% of all term newborns c-sections performed in our hospital. We excluded 5 videos due to congenital malformations. Videos had a total duration of 2,275 minutes [median of 10 (range 3-50) minutes per video]. DR-management in these infants was performed by 14 different caregivers with a frequency of 1 (1-101) patient’s per caregiver.

### Management in infants without any support of transition

The majority of infants (73%) did not receive any support of transition. In these infants, DR-management was analyzed for a median time of 10 minutes (range 3-10).

#### Prevention of heat loss

All but 3 infants were initially placed under the radiant warmer in a towel and were dried. The towel was replaced by a clean and warm one after 38 sec (4-156). In 18 cases towels were changed twice and in one infant 3 times. Temperature was measured in only 4 infants after 6 (4 – 9) minutes.

#### Assessment of infant’s condition

Auscultation was performed in all newborns, with the first auscultation occurring after 24 (2 – 179) seconds. A pulsoximetry sensor was placed at a median of 53 (16-240) seconds.

#### Other interventions

The umbilical cord was shortened in all but 3 infants after 109 (35-583) seconds. 16 infants (12%) were suctioned in median 2 (1-4) times lasting 9 (4-45) seconds per attempt. Newborns remained without any manipulation for 129 (2-373) seconds, that equals a 24 (0-69)% of the analyzed time. 22% (n = 30) of the infants were intermittently removed from under the radiant heater for 23 (14-389) seconds – mainly for weighing.

### Management in infants with support of postnatal transition

A total of 51 (27%) infants received a medical intervention. 46 (25%) received respiratory support via face mask and 17 of these were transferred to the NICU (12 for respiratory support, 5 for further observation). 5 infants without any respiratory support were transferred to the neonatal unit for other reasons (i.e. suspected infection). Management of infants with support of transition was analyzed for 18 (8-50) minutes.

#### Prevention of heat loss

**Figure 1:**
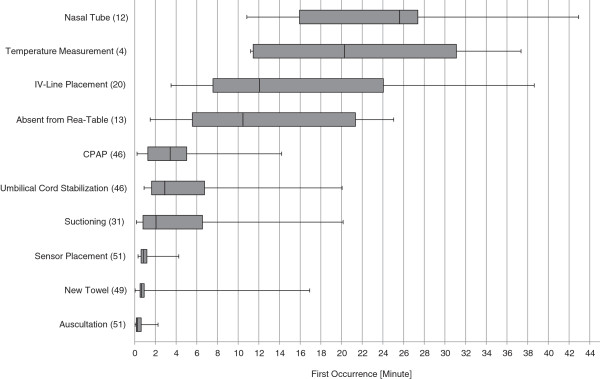
**First occurrence of various interventions.** Shown are how often different interventions were given (n in parenthesis). For each intervention the time point of first occurrence is given as median (horizontal line in the middle of each box), 25th and 75th percentiles (top and bottom of each box) and minimum/maximum (whiskers mark) of all analyzed recordings.

All except 2 infants were dried after arrival under the radiant heater and placed in a new towel. Time interval until placement into new towel varied significantly (Figure 1). Body temperature was measured in only 4 infants.

#### Assessment of infant’s condition

Auscultation was performed in all infants; a sensor for oxygen saturation was placed after a median of 52 seconds (Figure 1).

#### Suctioning

31 infants were suctioned in a median of 3 times (Table 1); total time spend for suctioning varied between 1 and 154 seconds, equaling between 0.1 and 8.7% of the analyzed time. In infants receiving respiratory support, first suctioning was performed mainly after CPAP administration (53%).

**Table 1:** **Duration of different interventions**^**a**^

**Intervention**	**n**	**Duration**	**% of analyzed time**
Respiratory support via mask	46	8.8 (1.5-28.5) min	46.9 (15.8-97.6)^b^
Sustained inflations	15	8.5 (1.8–13.8) s	2.6(0.2-8.4)^c^
Mechanical ventilation	7	53.1 (6.9-128.5) s	11.6(0.5-69.8)^c^
Suctioning	31	34.6 (0.9-153.6) s	3.2(0.1-8.7)^b^
CPAP prior to nasopharyngeal tube	12	22.6 (10-39.4) min^c^	
Stabilization prior to transport	22	27.3 (11.7-50) min^d^	

Given are number of infants receiving each intervention (n), the total time the intervention was administered (Total duration, given as median and range of all analyzed recordings) and the percentage of time spent for the intervention compared to total analyzed time (Relative duration, given as median and range of all recordings).

Abbreviations: min = minutes, s = seconds, CPAP = Continuous Positive Airway Pressure.

^a^Data are displayed as median and range.

^b^Percentage of total time that newborn was analyzed.

^c^Percentage of total mask application.

^d^Duration from arrival at the reanimation table until transfer to NICU (only infants that were transferred to NICU).

#### Respiratory support

Respiratory support via facemask was started between 0.2 and 14.2 minutes after arrival under radiant heater (Figure 1). Respiratory support was administered for a median duration of 8.8 minutes (Table 1).

A sustained inflation was performed in 15 infants (Table 1), with a median duration of a single sustained inflation of 8 (2-14) seconds. 7 infants received more than one sustained inflation. First sustained inflation was performed in median after 92 seconds and the last after 213 seconds.

Mechanical ventilation via face mask was administered in 7 infants for a median of 53 seconds. The team started PPV after auscultation in one case. In another one case suctioning was performed before PPV was started. Face mask was replaced by a naso-pharyngeal tube after 23 (10 - 39) minutes to administer CPAP for subsequent transport to the NICU. Time needed to place the nasopharyngeal tube was between 12 and 43 seconds.

Only 15 out of 46 patients received CPAP without any interruption. In the majority of infants (74%), respiratory problems resolved after CPAP administration for about 6.5 (1.5-28.5) minutes.

#### IV-line placement

An IV-line was placed in 20 infants after 12 minutes (Figure 1). In 8 infants several attempts [3 (2-4)] were required until successful placement. Total time required until an IV-line was successfully placed was 6.9 (3.6 – 13.9) minutes corresponding to 23% of the analyzed time. Time needed only for the successful attempt was 5 minutes (2.9-8.6).

### Sequence of interventions

**Figure 2:**
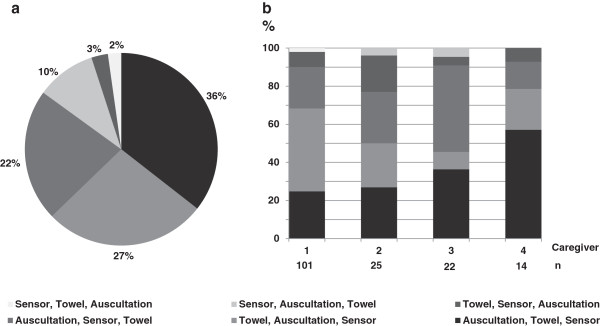
**Proportions of sequences of interventions. a)** Pie chart showing different sequences and their percentage of the total video analyses. **b)** Vertical bar chart showing percentage of different sequences regarding 4 different caregivers.

The first intervention that occurred in most of the infants was auscultation (104 infants), towel changing (54) and sensor placement (23). The most frequent sequence of these three interventions is shown in Figure 2.The majority of infants were handled by 4 different caregivers. Data analysis showed that each caregiver had a preference with respect to the sequence of interventions; however caregivers did not adhere to their personal preference in about 40% of the infants (Figure 2).

## Discussion

Several studies describe current practice in newborn resuscitation, which is required only in a small number of infants. The majority of term infants do not need any intervention at all; a few infants require just some support of postnatal transition. However, only little is known regarding current practice of supporting postnatal transition.

To the best of our knowledge, we describe for the first time current practice of care in a large number of term infants born by c-section in a single center. The study provides data for an internal quality improvement program and allows benchmarking with current practice in other centers. Furthermore, our study supports the benefits of using routine video recording of DR-management.

In our low-risk population of term infants born by c-section one out of four postnatal transition was supported. Most of these infants were initially vigorous and signs of respiratory distress became prominent after 3 minutes. Less than 7 minutes of CPAP administration were sufficient to prevent a NICU-admission in about 75% of these infants. Whereas previous cohort studies have already described the need for respiratory support and subsequent NICU admission in term infants delivered by c-section [4,10], little was known regarding the time course of interventions in the delivery room. According to our data, sufficient support of respiratory transition within a few minutes after birth will prevent the need of NICU admission, supporting the need of immediate pediatric expertise after c-section even in a low risk population.

Video recording is part of a quality assurance program in our institution. To our opinion, video recording represents the only valid tool to assess current practice in DR-management [11,12], since other methods like surveys or structured direct observations do have severe limitations [13-15]. As a consequence of the current analysis, DR-management was questioned and medical staff was trained concerning the following issues. Sustained inflation: 15 infants received sustained inflation, which should be administered for 10 seconds if heart rate is below 100 bpm [16]. In the analyzed patients duration of sustained inflation ranged between 2 and 14 seconds and the last was administered seven minutes after placement under radiant heater in an infant which has cried before. Since the only evidence for a benefit of sustained inflation is available for preterm infants without any spontaneous breathing [16], we emphasized the very restricted use of sustained inflations. CPAP-administration: CPAP administration was interrupted in some infants since hands of caregiver were needed for other interventions. Since CPAP is known to be effective mainly if administered constantly [17], we recommend the use of naso-pharyngeal tube (which has been shown as effective as masks for CPAP administration) if continuous mask administration is not feasible [18,19]. Interestingly, a median of 24 seconds was required to place nasopharyngeal tube in the present study, thus we do not recommend that method as first line intervention in infants with severe respiratory problems. Suctioning: Whereas suctioning was recommended in old guidelines and textbooks, it is nowadays considered unnecessary and potentially harmful and was deleted in internal guidelines [1,20]. Nevertheless, in our analysis about 25% of infants were suctioned and total time spend for suctioning varied but lasted up to three minutes. Thus, teaching of junior staff focusses on that issue and new results regarding the benefit of just wiping the mouth are presented [21].

Some other aspects of our analysis are of interest for clinical practice. Peripheral line placement represents a common procedure in neonatology. To our knowledge no systematic analysis was performed previously to investigate time needed to place a line in term infants. In the current analysis 20 infants received a peripheral venous line; in 40% more than one attempt was needed. In stable term infants without any cardio-circular problems total time spend for placing the venous access varied between 3 and 14 minutes. These results support the danger of time-loss if trying to place a iv-access in unstable newborns during resuscitation. However, little is known regarding the time required to place umbilical cord or intraosseous access.

Finally, our data are of interest with respect to improving team work – an important aspect of neonatal care. It is known that quality of team work can be improved if caregivers adhere to a standardized sequence of interventions. Interestingly, even the sequence of three very basic interventions varied between and within different caregivers. Whereas it could be argued, that sequence depended on condition of the individual infant, varying order of intervention could still become a problem during treatment of more complex patients (such as very preterm infants) which require a team of varying caregivers.

Whereas the analysis provides interesting data regarding care of low-risk term infants after c-section, some limitations have to be discussed. Firstly, the study did not intend to investigate short or long term outcome of the infants. Thus, no conclusions can be drawn regarding efficacy of DR-management. Secondly, this study was not designed to investigate the reasons of a disturbed postnatal adaptation or to verify the indication for interventions. It was the intention to describe current practice. Finally, the analysis is single center based and thus conclusions are not always generalizable. Nevertheless, we consider some of the findings thought-provoking.

## Conclusions

In summary, this study highlights the usefulness of video recording of the DR-management and presents detailed analysis of video recordings of support of postnatal transition in a low-risk population of term infants after c-section. The data can be used for benchmarking purposes between different institutions and as an example on how to improve management in delivery room.

## Abbreviations

DR: Delivery room.

## Competing interests

The authors declare that they have no competing interests.

## Authors’ contributions

DK and MR conceptualized and designed the study, drafted the initial manuscript, carried out initial analyses, contributed to the acquisition of data. SI, JD and WB contributed to the design of the study and for acquisition of data. All authors read and approved the final manuscript.

## Pre-publication history

The pre-publication history for this paper can be accessed here:

http://www.biomedcentral.com/1471-2393/14/225/prepub
